# Dimethyl­bis­(2-methyl­quinolin-8-olato-κ^2^ *N*,*O*)tin(IV)

**DOI:** 10.1107/S1600536812047393

**Published:** 2012-11-28

**Authors:** Ezzatollah Najafi, Mostafa M. Amini, Seik Weng Ng

**Affiliations:** a Department of Chemistry, General Campus, Shahid Beheshti University, Tehran 1983963113, Iran; b Department of Chemistry, University of Malaya, 50603 Kuala Lumpur, Malaysia; c Chemistry Department, Faculty of Science, King Abdulaziz University, PO Box 80203 Jeddah, Saudi Arabia

## Abstract

The Sn^IV^ cation in the title compound, [Sn(CH_3_)_2_(C_10_H_8_NO)_2_], is *N*,*O*-chelated by two 2-methyl­quinolin-8-olate anions and coordinated by two methyl groups in a skew-trapezoidal bipyramidal geometry. In the mol­ecule, the two quinoline ring systems are twisted to one another at 10.91 (18)°. The dimethyl­tin skeleton [C—Sn—C = 149.6 (2)°] is bent over the longer edge of the trapezoid that is defined by the four chelating atoms. Weak inter­molecular C—H⋯O hydrogen bonding occurs in the crystal.

## Related literature

For ethyl­propyl­bis­(2-methyl-8-quinolinato)tin(IV), see: Das *et al.* (1984 ▶).
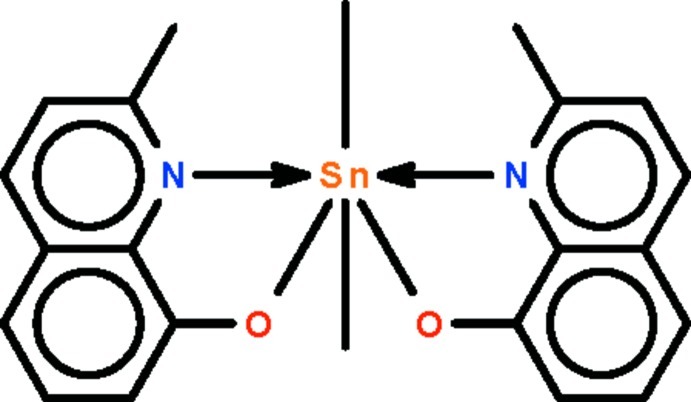


## Experimental

### 

#### Crystal data

[Sn(CH_3_)_2_(C_10_H_8_NO)_2_]
*M* *_r_* = 465.11
Monoclinic, 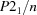

*a* = 8.0434 (4) Å
*b* = 20.6952 (10) Å
*c* = 12.0102 (6) Å
β = 95.420 (5)°
*V* = 1990.28 (17) Å^3^
*Z* = 4
Mo *K*α radiation
μ = 1.30 mm^−1^
*T* = 295 K
0.25 × 0.25 × 0.05 mm

#### Data collection

Agilent SuperNova Dual diffractometer with an Atlas detector
Absorption correction: multi-scan (*CrysAlis PRO*; Agilent, 2012 ▶) *T*_min_ = 0.737, *T*_max_ = 0.938
20963 measured reflections
4600 independent reflections
3410 reflections with *I* > 2σ(*I*)
*R*_int_ = 0.046

#### Refinement

*R*[*F* ^2^ > 2σ(*F* ^2^)] = 0.039
*wR*(*F* ^2^) = 0.094
*S* = 1.05
4600 reflections
246 parameters
30 restraints
H-atom parameters constrained
Δρ_max_ = 0.79 e Å^−3^
Δρ_min_ = −0.49 e Å^−3^

### 

Data collection: *CrysAlis PRO* (Agilent, 2012 ▶); cell refinement: *CrysAlis PRO*; data reduction: *CrysAlis PRO*; program(s) used to solve structure: *SHELXS97* (Sheldrick, 2008 ▶); program(s) used to refine structure: *SHELXL97* (Sheldrick, 2008 ▶); molecular graphics: *X-SEED* (Barbour, 2001 ▶); software used to prepare material for publication: *publCIF* (Westrip, 2010 ▶).

## Supplementary Material

Crystal structure: contains datablock(s) global, I. DOI: 10.1107/S1600536812047393/xu5653sup1.cif

Structure factors: contains datablock(s) I. DOI: 10.1107/S1600536812047393/xu5653Isup2.hkl

Additional supplementary materials:  crystallographic information; 3D view; checkCIF report

## Figures and Tables

**Table 1:** Hydrogen-bond geometry (Å, °)

*D*—H⋯*A*	*D*—H	H⋯*A*	*D*⋯*A*	*D*—H⋯*A*
C3—H3*C*⋯O2^i^	0.96	2.49	3.353 (7)	149

Symmetry code: (i) 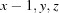.

**Fig. 1.**
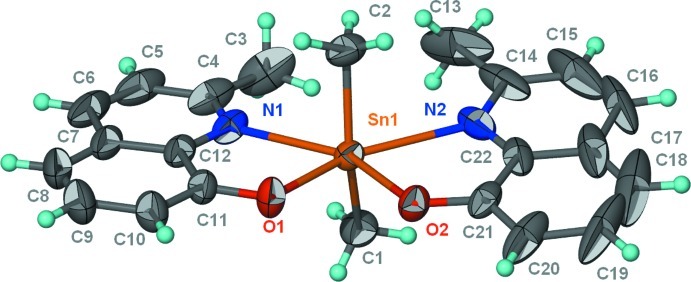
Thermal ellipsoid plot (Barbour, 2001) of (CH3)2Sn(C10H8NO)2 at the 50% probability level; hydrogen atoms are drawn as spheres of arbitrary radius.

## supplementary crystallographic information

### Comment

The deprotonated 2-methy-8-hydroxyquinoline ligand chelates to the metal atom of a diorganotin skeleton bu the proximity of the methyl substitutent in the aromatic system results in a six-coordinate geometry that is distorted towards a skew-trapezoidal bipyramid, as noted in the ethylpropyltin derivative (Kumar Das *et al.*, 1984). The Sn^IV^ atom in the dimethyltin analog (Scheme I, Fig. 1) is chelated by the 2-methyl-8-quinolinate ion and it exists in a skew-trapezoidal bipyramidal geometry [C–Sn–C 149.6 (2) °].

The dimethyltin skeleton is arched over the long side of the trazepoid defined by the chelating N and O atoms.

### Experimental

Dimethyltin dichloride (0.22 g, 1 mmol) and 2-methyl-8-hydroxyquinoline (0.36 g, 2 mmol) were loaded into a convection tube; the tube was filled with ethyl alcohol and kept at 333 K. Yellow crystals were collected from the side arm after several days.

### Refinement

Carbon-bound H-atoms were placed in calculated positions [C–H 0.93 to 0.96 Å, *U*_iso_(H) 1.2 to 1.5*U*_eq_(C)] and were included in the refinement in the riding model approximation.

As the atoms C15 to C19 showed somewhat elongated ellipsoids, their anisotropic temperature factors were restrained to approximate isotropic behavior.

### Crystal data

**Table:**

[Sn(CH_3_)_2_(C_10_H_8_NO)_2_]	*F*(000) = 936
*M**_r_* = 465.11	*D*_x_ = 1.552 Mg m^−^^3^
Monoclinic, *P*2_1_/*n*	Mo *K*α radiation, λ = 0.71073 Å
Hall symbol: -P 2yn	Cell parameters from 4858 reflections
*a* = 8.0434 (4) Å	θ = 2.9–27.5°
*b* = 20.6952 (10) Å	µ = 1.30 mm^−^^1^
*c* = 12.0102 (6) Å	*T* = 295 K
β = 95.420 (5)°	Prism, yellow
*V* = 1990.28 (17) Å^3^	0.25 × 0.25 × 0.05 mm
*Z* = 4	

### Data collection

**Table:**

Agilent SuperNova Dual diffractometer with an Atlas detector	4600 independent reflections
Radiation source: SuperNova (Mo) X-ray Source	3410 reflections with *I* > 2σ(*I*)
Mirror monochromator	*R*_int_ = 0.046
Detector resolution: 10.4041 pixels mm^-1^	θ_max_ = 27.6°, θ_min_ = 2.9°
ω scan	*h* = −10→10
Absorption correction: multi-scan (*CrysAlis PRO*; Agilent, 2012)	*k* = −26→26
*T*_min_ = 0.737, *T*_max_ = 0.938	*l* = −11→15
20963 measured reflections	

### Refinement

**Table:**

Refinement on *F*^2^	Primary atom site location: structure-invariant direct methods
Least-squares matrix: full	Secondary atom site location: difference Fourier map
*R*[*F*^2^ > 2σ(*F*^2^)] = 0.039	Hydrogen site location: inferred from neighbouring sites
*wR*(*F*^2^) = 0.094	H-atom parameters constrained
*S* = 1.05	*w* = 1/[σ^2^(*F*_o_^2^) + (0.0354*P*)^2^ + 1.6968*P*] where *P* = (*F*_o_^2^ + 2*F*_c_^2^)/3
4600 reflections	(Δ/σ)_max_ = 0.001
246 parameters	Δρ_max_ = 0.79 e Å^−^^3^
30 restraints	Δρ_min_ = −0.49 e Å^−^^3^

### Fractional atomic coordinates and isotropic or equivalent isotropic displacement parameters (Å^2^)

**Table:**

	*x*	*y*	*z*	*U*_iso_*/*U*_eq_	
Sn1	0.31642 (3)	0.682497 (12)	0.39615 (2)	0.04048 (10)	
O1	0.4151 (3)	0.60834 (13)	0.3067 (3)	0.0537 (7)	
O2	0.5350 (3)	0.72786 (13)	0.3645 (2)	0.0511 (7)	
N1	0.1010 (4)	0.59118 (17)	0.3762 (3)	0.0524 (9)	
N2	0.3308 (6)	0.79119 (19)	0.4985 (3)	0.0644 (11)	
C1	0.1501 (6)	0.7308 (2)	0.2773 (4)	0.0610 (12)	
H1A	0.1756	0.7195	0.2033	0.091*	
H1B	0.1618	0.7767	0.2876	0.091*	
H1C	0.0376	0.7183	0.2871	0.091*	
C3	−0.1319 (7)	0.6398 (3)	0.4603 (6)	0.099 (2)	
H3A	−0.0620	0.6773	0.4589	0.149*	
H3B	−0.1453	0.6285	0.5365	0.149*	
H3C	−0.2392	0.6489	0.4216	0.149*	
C2	0.3722 (6)	0.6462 (2)	0.5594 (4)	0.0641 (13)	
H2A	0.3911	0.6005	0.5561	0.096*	
H2B	0.2802	0.6546	0.6028	0.096*	
H2C	0.4707	0.6671	0.5937	0.096*	
C4	−0.0528 (6)	0.5848 (3)	0.4043 (4)	0.0696 (15)	
C5	−0.1415 (7)	0.5271 (4)	0.3834 (5)	0.085 (2)	
H5	−0.2499	0.5234	0.4037	0.102*	
C6	−0.0701 (9)	0.4767 (3)	0.3338 (5)	0.093 (2)	
H6	−0.1293	0.4384	0.3215	0.112*	
C7	0.0916 (7)	0.4816 (2)	0.3008 (4)	0.0701 (16)	
C8	0.1728 (11)	0.4328 (3)	0.2467 (5)	0.095 (2)	
H8	0.1207	0.3931	0.2323	0.114*	
C9	0.3267 (11)	0.4433 (3)	0.2156 (5)	0.098 (2)	
H9	0.3788	0.4105	0.1790	0.117*	
C10	0.4118 (7)	0.5019 (2)	0.2362 (4)	0.0684 (14)	
H10	0.5186	0.5072	0.2140	0.082*	
C11	0.3376 (6)	0.5519 (2)	0.2895 (4)	0.0511 (10)	
C12	0.1746 (5)	0.5415 (2)	0.3235 (4)	0.0510 (11)	
C13	0.0724 (11)	0.7858 (4)	0.5910 (6)	0.134 (3)	
H13A	0.0170	0.8107	0.6438	0.201*	
H13B	0.0992	0.7438	0.6218	0.201*	
H13C	0.0002	0.7811	0.5231	0.201*	
C14	0.2287 (10)	0.8192 (3)	0.5665 (4)	0.096 (2)	
C15	0.2632 (14)	0.8788 (4)	0.6124 (6)	0.125 (3)	
H15	0.1897	0.8978	0.6580	0.150*	
C16	0.3987 (12)	0.9086 (3)	0.5919 (6)	0.106 (3)	
H16	0.4212	0.9485	0.6255	0.127*	
C17	0.5152 (11)	0.8834 (3)	0.5205 (6)	0.100 (2)	
C18	0.6547 (14)	0.9089 (4)	0.4912 (8)	0.132 (3)	
H18	0.6857	0.9488	0.5225	0.159*	
C19	0.7582 (10)	0.8829 (4)	0.4195 (8)	0.117 (3)	
H19	0.8523	0.9053	0.4014	0.140*	
C20	0.7173 (7)	0.8171 (3)	0.3703 (6)	0.095 (2)	
H20	0.7846	0.7974	0.3213	0.114*	
C21	0.5752 (6)	0.7875 (2)	0.4016 (4)	0.0576 (13)	
C22	0.4726 (8)	0.8203 (2)	0.4745 (4)	0.0658 (15)	

### Atomic displacement parameters (Å^2^)

**Table:**

	*U*^11^	*U*^22^	*U*^33^	*U*^12^	*U*^13^	*U*^23^
Sn1	0.03944 (16)	0.03883 (16)	0.04375 (17)	0.00020 (12)	0.00699 (11)	0.00239 (12)
O1	0.0472 (16)	0.0403 (16)	0.076 (2)	−0.0065 (13)	0.0160 (15)	−0.0110 (14)
O2	0.0461 (16)	0.0464 (17)	0.0624 (18)	−0.0095 (13)	0.0135 (14)	−0.0011 (14)
N1	0.0410 (19)	0.052 (2)	0.063 (2)	−0.0057 (16)	−0.0012 (17)	0.0181 (18)
N2	0.099 (3)	0.053 (2)	0.039 (2)	0.023 (2)	−0.006 (2)	−0.0055 (18)
C1	0.060 (3)	0.058 (3)	0.062 (3)	0.004 (2)	−0.005 (2)	0.009 (2)
C3	0.055 (3)	0.110 (5)	0.138 (6)	0.022 (3)	0.034 (4)	0.050 (5)
C2	0.067 (3)	0.068 (3)	0.056 (3)	−0.004 (2)	0.002 (2)	0.020 (2)
C4	0.041 (2)	0.089 (4)	0.078 (3)	−0.010 (3)	−0.004 (2)	0.037 (3)
C5	0.053 (3)	0.118 (5)	0.079 (4)	−0.036 (3)	−0.013 (3)	0.047 (4)
C6	0.097 (5)	0.103 (5)	0.071 (4)	−0.065 (4)	−0.029 (4)	0.038 (4)
C7	0.093 (4)	0.058 (3)	0.053 (3)	−0.034 (3)	−0.021 (3)	0.014 (2)
C8	0.159 (7)	0.053 (3)	0.068 (4)	−0.040 (4)	−0.013 (4)	−0.005 (3)
C9	0.162 (7)	0.050 (3)	0.078 (4)	−0.007 (4)	−0.001 (4)	−0.018 (3)
C10	0.088 (4)	0.047 (3)	0.069 (3)	0.002 (3)	0.004 (3)	−0.010 (2)
C11	0.062 (3)	0.038 (2)	0.052 (2)	−0.001 (2)	−0.002 (2)	−0.0003 (19)
C12	0.055 (3)	0.046 (2)	0.049 (2)	−0.010 (2)	−0.010 (2)	0.011 (2)
C13	0.144 (7)	0.190 (8)	0.077 (4)	0.086 (7)	0.060 (5)	0.019 (5)
C14	0.160 (7)	0.085 (4)	0.040 (3)	0.066 (4)	−0.009 (4)	−0.014 (3)
C15	0.174 (7)	0.116 (6)	0.080 (4)	0.063 (5)	−0.015 (5)	−0.021 (4)
C16	0.172 (6)	0.062 (4)	0.072 (4)	0.042 (4)	−0.045 (4)	−0.032 (3)
C17	0.132 (5)	0.067 (4)	0.089 (4)	0.000 (4)	−0.054 (4)	0.010 (3)
C18	0.149 (7)	0.094 (5)	0.139 (6)	−0.020 (5)	−0.067 (6)	0.026 (5)
C19	0.086 (4)	0.097 (5)	0.156 (6)	−0.053 (4)	−0.045 (4)	0.062 (4)
C20	0.057 (3)	0.077 (4)	0.143 (6)	−0.022 (3)	−0.025 (4)	0.053 (4)
C21	0.054 (3)	0.049 (3)	0.065 (3)	−0.013 (2)	−0.018 (2)	0.023 (2)
C22	0.100 (4)	0.037 (2)	0.053 (3)	0.007 (3)	−0.033 (3)	−0.004 (2)

### Geometric parameters (Å, º)

**Table:**

Sn1—O2	2.060 (3)	C7—C8	1.397 (9)
Sn1—O1	2.073 (3)	C7—C12	1.422 (6)
Sn1—C2	2.108 (4)	C8—C9	1.344 (10)
Sn1—C1	2.113 (4)	C8—H8	0.9300
Sn1—N1	2.560 (3)	C9—C10	1.403 (8)
Sn1—N2	2.561 (4)	C9—H9	0.9300
O1—C11	1.331 (5)	C10—C11	1.381 (6)
O2—C21	1.341 (5)	C10—H10	0.9300
N1—C4	1.320 (6)	C11—C12	1.426 (6)
N1—C12	1.369 (6)	C13—C14	1.489 (11)
N2—C14	1.343 (7)	C13—H13A	0.9600
N2—C22	1.345 (7)	C13—H13B	0.9600
C1—H1A	0.9600	C13—H13C	0.9600
C1—H1B	0.9600	C14—C15	1.368 (10)
C1—H1C	0.9600	C15—C16	1.296 (11)
C3—C4	1.495 (8)	C15—H15	0.9300
C3—H3A	0.9600	C16—C17	1.427 (11)
C3—H3B	0.9600	C16—H16	0.9300
C3—H3C	0.9600	C17—C18	1.318 (12)
C2—H2A	0.9600	C17—C22	1.448 (7)
C2—H2B	0.9600	C18—C19	1.364 (12)
C2—H2C	0.9600	C18—H18	0.9300
C4—C5	1.400 (8)	C19—C20	1.508 (10)
C5—C6	1.356 (9)	C19—H19	0.9300
C5—H5	0.9300	C20—C21	1.380 (7)
C6—C7	1.399 (9)	C20—H20	0.9300
C6—H6	0.9300	C21—C22	1.430 (8)
			
O2—Sn1—O1	82.34 (11)	C8—C7—C12	119.3 (5)
O2—Sn1—C2	102.92 (16)	C6—C7—C12	116.1 (6)
O1—Sn1—C2	99.20 (17)	C9—C8—C7	119.6 (5)
O2—Sn1—C1	99.00 (16)	C9—C8—H8	120.2
O1—Sn1—C1	104.44 (16)	C7—C8—H8	120.2
C2—Sn1—C1	149.6 (2)	C8—C9—C10	122.6 (6)
O2—Sn1—N1	154.31 (12)	C8—C9—H9	118.7
O1—Sn1—N1	72.16 (12)	C10—C9—H9	118.7
C2—Sn1—N1	84.66 (15)	C11—C10—C9	120.3 (6)
C1—Sn1—N1	84.63 (15)	C11—C10—H10	119.9
O2—Sn1—N2	71.80 (14)	C9—C10—H10	119.9
O1—Sn1—N2	153.73 (14)	O1—C11—C10	120.8 (4)
C2—Sn1—N2	82.55 (16)	O1—C11—C12	121.2 (4)
C1—Sn1—N2	84.58 (15)	C10—C11—C12	118.0 (4)
N1—Sn1—N2	133.87 (15)	N1—C12—C7	121.8 (5)
C11—O1—Sn1	122.2 (3)	N1—C12—C11	117.9 (4)
C21—O2—Sn1	122.8 (3)	C7—C12—C11	120.2 (5)
C4—N1—C12	120.0 (4)	C14—C13—H13A	109.5
C4—N1—Sn1	133.9 (4)	C14—C13—H13B	109.5
C12—N1—Sn1	106.2 (3)	H13A—C13—H13B	109.5
C14—N2—C22	121.1 (5)	C14—C13—H13C	109.5
C14—N2—Sn1	132.0 (5)	H13A—C13—H13C	109.5
C22—N2—Sn1	106.9 (3)	H13B—C13—H13C	109.5
Sn1—C1—H1A	109.5	N2—C14—C15	121.4 (9)
Sn1—C1—H1B	109.5	N2—C14—C13	119.7 (6)
H1A—C1—H1B	109.5	C15—C14—C13	118.8 (7)
Sn1—C1—H1C	109.5	C16—C15—C14	119.6 (9)
H1A—C1—H1C	109.5	C16—C15—H15	120.2
H1B—C1—H1C	109.5	C14—C15—H15	120.2
C4—C3—H3A	109.5	C15—C16—C17	123.5 (7)
C4—C3—H3B	109.5	C15—C16—H16	118.2
H3A—C3—H3B	109.5	C17—C16—H16	118.2
C4—C3—H3C	109.5	C18—C17—C16	129.7 (8)
H3A—C3—H3C	109.5	C18—C17—C22	115.9 (9)
H3B—C3—H3C	109.5	C16—C17—C22	114.5 (7)
Sn1—C2—H2A	109.5	C17—C18—C19	126.6 (9)
Sn1—C2—H2B	109.5	C17—C18—H18	116.7
H2A—C2—H2B	109.5	C19—C18—H18	116.7
Sn1—C2—H2C	109.5	C18—C19—C20	118.7 (7)
H2A—C2—H2C	109.5	C18—C19—H19	120.6
H2B—C2—H2C	109.5	C20—C19—H19	120.6
N1—C4—C5	120.9 (6)	C21—C20—C19	116.7 (7)
N1—C4—C3	119.2 (5)	C21—C20—H20	121.6
C5—C4—C3	119.9 (5)	C19—C20—H20	121.6
C6—C5—C4	120.3 (6)	O2—C21—C20	119.9 (5)
C6—C5—H5	119.9	O2—C21—C22	120.3 (4)
C4—C5—H5	119.9	C20—C21—C22	119.8 (5)
C5—C6—C7	120.9 (5)	N2—C22—C21	118.0 (4)
C5—C6—H6	119.6	N2—C22—C17	119.8 (6)
C7—C6—H6	119.6	C21—C22—C17	122.2 (6)
C8—C7—C6	124.6 (6)		
			
O2—Sn1—O1—C11	178.2 (3)	Sn1—O1—C11—C12	5.6 (5)
C2—Sn1—O1—C11	76.3 (3)	C9—C10—C11—O1	−178.1 (5)
C1—Sn1—O1—C11	−84.4 (3)	C9—C10—C11—C12	0.8 (7)
N1—Sn1—O1—C11	−5.0 (3)	C4—N1—C12—C7	−2.0 (6)
N2—Sn1—O1—C11	168.1 (3)	Sn1—N1—C12—C7	178.5 (3)
O1—Sn1—O2—C21	−178.9 (3)	C4—N1—C12—C11	176.8 (4)
C2—Sn1—O2—C21	−81.2 (3)	Sn1—N1—C12—C11	−2.8 (4)
C1—Sn1—O2—C21	77.6 (3)	C8—C7—C12—N1	179.9 (4)
N1—Sn1—O2—C21	174.0 (3)	C6—C7—C12—N1	0.9 (6)
N2—Sn1—O2—C21	−3.6 (3)	C8—C7—C12—C11	1.2 (7)
O2—Sn1—N1—C4	−168.1 (4)	C6—C7—C12—C11	−177.8 (4)
O1—Sn1—N1—C4	−175.5 (4)	O1—C11—C12—N1	−1.0 (6)
C2—Sn1—N1—C4	83.0 (4)	C10—C11—C12—N1	−179.9 (4)
C1—Sn1—N1—C4	−68.5 (4)	O1—C11—C12—C7	177.8 (4)
N2—Sn1—N1—C4	8.7 (5)	C10—C11—C12—C7	−1.1 (6)
O2—Sn1—N1—C12	11.4 (4)	C22—N2—C14—C15	1.2 (8)
O1—Sn1—N1—C12	4.0 (2)	Sn1—N2—C14—C15	179.9 (4)
C2—Sn1—N1—C12	−97.5 (3)	C22—N2—C14—C13	179.8 (5)
C1—Sn1—N1—C12	111.0 (3)	Sn1—N2—C14—C13	−1.5 (7)
N2—Sn1—N1—C12	−171.8 (2)	N2—C14—C15—C16	−1.4 (10)
O2—Sn1—N2—C14	−177.6 (4)	C13—C14—C15—C16	−180.0 (7)
O1—Sn1—N2—C14	−167.0 (4)	C14—C15—C16—C17	1.7 (12)
C2—Sn1—N2—C14	−71.3 (4)	C15—C16—C17—C18	178.6 (8)
C1—Sn1—N2—C14	81.1 (4)	C15—C16—C17—C22	−1.7 (9)
N1—Sn1—N2—C14	3.9 (5)	C16—C17—C18—C19	−177.9 (7)
O2—Sn1—N2—C22	1.3 (3)	C22—C17—C18—C19	2.4 (11)
O1—Sn1—N2—C22	11.9 (4)	C17—C18—C19—C20	−2.6 (12)
C2—Sn1—N2—C22	107.6 (3)	C18—C19—C20—C21	0.2 (9)
C1—Sn1—N2—C22	−100.0 (3)	Sn1—O2—C21—C20	−175.0 (3)
N1—Sn1—N2—C22	−177.2 (2)	Sn1—O2—C21—C22	5.7 (5)
C12—N1—C4—C5	1.4 (7)	C19—C20—C21—O2	−177.4 (4)
Sn1—N1—C4—C5	−179.1 (3)	C19—C20—C21—C22	1.9 (7)
C12—N1—C4—C3	−179.2 (4)	C14—N2—C22—C21	179.9 (4)
Sn1—N1—C4—C3	0.3 (7)	Sn1—N2—C22—C21	0.9 (4)
N1—C4—C5—C6	0.1 (8)	C14—N2—C22—C17	−1.2 (7)
C3—C4—C5—C6	−179.3 (5)	Sn1—N2—C22—C17	179.7 (3)
C4—C5—C6—C7	−1.2 (8)	O2—C21—C22—N2	−4.0 (6)
C5—C6—C7—C8	−178.3 (5)	C20—C21—C22—N2	176.7 (4)
C5—C6—C7—C12	0.6 (7)	O2—C21—C22—C17	177.2 (4)
C6—C7—C8—C9	178.0 (6)	C20—C21—C22—C17	−2.1 (7)
C12—C7—C8—C9	−0.9 (8)	C18—C17—C22—N2	−178.8 (5)
C7—C8—C9—C10	0.7 (10)	C16—C17—C22—N2	1.4 (7)
C8—C9—C10—C11	−0.6 (9)	C18—C17—C22—C21	0.0 (8)
Sn1—O1—C11—C10	−175.5 (3)	C16—C17—C22—C21	−179.8 (4)

### Hydrogen-bond geometry (Å, º)

**Table:**

*D*—H···*A*	*D*—H	H···*A*	*D*···*A*	*D*—H···*A*
C3—H3*C*···O2^i^	0.96	2.49	3.353 (7)	149

Symmetry code: (i) *x*−1, *y*, *z*.
